# Efficacy of vitamin D supplementation on the severity of atopic dermatitis in children: A systematic review and meta-analysis

**DOI:** 10.12688/f1000research.106957.2

**Published:** 2023-09-25

**Authors:** Afif Nurul Hidayati, Sawitri Sawitri, Desiana Widityaning Sari, Cita Rosita Sigit Prakoeswa, Diah Mira Indramaya, Damayanti Damayanti, Iskandar Zulkarnain, Irmadita Citrashanty, Yuri Widia, Sylvia Anggraeni

**Affiliations:** 1 Department of Dermatology and Venereology, Faculty of Medicine, Dr. Soetomo Hospital, Surabaya, East Java, 60132, Indonesia; 2 Department of Dermatology and Venereology, Universitas Airlangga Hospital, Surabaya, East Java, 60115, Indonesia

**Keywords:** atopic dermatitis, children, efficacy, vitamin D, human health

## Abstract

**Background:** Atopic Dermatitis (AD) is a common dermatosis in children, that includes skin architecture defects, immune dysregulation, and changes of skin flora. Several new drugs have been found to reduce the severity of AD. Vitamin D is one of the new therapies that is still controversial. The purpose of this research is to conclude the efficacy of vitamin D on atopic dermatitis severity in children aged 0-18 years old.

**Methods:** A systematic search was conducted on the PubMed, Cochrane, ProQuest, Google Scholar, Clinical Trial website, and university repositories including studies published from January 2010 through October 2020. We compared populations, intervention, study design, and outcomes. Statistical analysis was done with Review Manager 5.4.1.

**Results:** Eight articles met eligibility and inclusion criteria, four articles provided complete data and were analysed. Not all studies demonstrated the efficacy of vitamin D but a meta-analysis of four studies of vitamin D supplementation vs placebo found a mean difference of -0.93 (95%CI -1.76, to -0.11, *p*<0.001) of patient outcome, but statistically, there was no difference in cure rate (risk ratio 1.46 (95%CI 0.72, to 2.97, *p*=0.008) in vitamin D supplementation groups compared to placebo groups.

**Conclusions:** Vitamin D supplementation in paediatric atopic dermatitis patients could offer improvement of disease severity but the recommended dose and duration of administration cannot be concluded yet.

## Introduction

Atopic Dermatitis (AD) is now considered a complex multifactorial disease that includes defects in skin barrier structures, immune dysregulation, genetic susceptibility, and changes in skin flora which mostly occur in infancy and childhood. Based on the clinical features, AD can be divided into 3 forms, namely AD in infants (2 months-2 years), children (2–12 years), and adolescents (over 12 years). ^1^ Increasing prevalence of AD has been reported in areas including the Asia-Pacific region, where it is reported that 88% of children with AD have either mild or moderate and 12% have severe AD. However, Indonesia still has a lower prevalence in children between 6-7 years old when compared to Thailand and Malaysia, and a lower prevalence in children aged 13-14 years when compared to Pakistan. ^2^

We found data on the prevalence of pediatric AD patients who had vitamin D insufficiency and deficiency in a study conducted by Wang *et al.*, ^3^ from 498 pediatric AD patients, 47.8% patients had vitamin D deficiency, 41% patients had vitamin D insufficiency and only 11.2% patients who have normal serum vitamin D levels. It was also found in a study conducted by Farazjadeh *et al.*, ^4^ that the average serum vitamin D level in pediatric AD patients was lower than controls. Although this study did not describe in detail the prevalence of patients with vitamin D deficiency and vitamin D insufficiency.

In addition to the reduction of skin inflammation, recently, AD treatment has focused more on the regulation of the immune response and enhancing the barrier function of the skin. ^5^ Poor compliance with the use of topical drugs makes some researchers try to find other drugs that are not only safe, cheap, easy to use but also effective. Several recent studies have shown that vitamin D supplementation may be an option, although the results of intervention trials are still conflicting. ^6^

In AD patients, defects in the skin barrier structure, as well as decreased functional integrity and reduced ability to regenerate have roles in inducing immune responses and specific inflammatory reactions. ^7^ In acute lesions, there will be a decrease in AMP (Antimicrobial Peptide) production, an increase in *S. aureus* colonization, and an effect on the severity of the disease and reduce the risk of infection. Vitamin D can increase barrier function, induce AMP and enhance monocyte and macrophage cell function. ^8^ Vitamin D has been known to have some effects on the innate and adaptive immune systems. Several mechanisms can modulate the progression of AD lesions, such as increasing epidermal differentiation, increasing production of cathelicidin, decreasing Th2 cytokines, decreasing Ig E production, decreasing B cell proliferation, and upregulating of T cells. ^9^

A previous systematic review and meta-analysis in 2019 on vitamin D and AD had reported a highly statistically significant reduction in SCORAD (Scoring of Atopic Dermatitis) on intervention with vitamin D, in the paediatric and adult population. ^10^ While a systematic review published by Huang on the paediatric AD population in 2018 concluded that 67% of the collected studies reported a significant improvement in AD severity with vitamin D supplementation, but this systematic review did not include a meta-analysis. ^11^ We conducted a meta-analysis with research published in last 10 years because there has been an increase in publications regarding vitamin D supplementation during this time. ^12^

The main objective of our systematic review and meta-analysis is to provide an updated review of the interventional study of vitamin D in the paediatric AD population to investigate clinical outcomes from measuring scales.

## Methods

### Search procedures

We conducted a systematic search of the literature on several databases, namely PubMed, Cochrane Library, ProQuest, and a clinical trial website, ClinicalTrials.gov with keywords showed in Table 1 (see also *Extended data* ^35^). We also did manual hand searching on Google Scholar and searched for grey literature on the repository (including research from January 1 ^st^ 2010 to October 31 ^st^ 2020, and the databases were last searched on 2 ^nd^ November 2020). The search procedure was based on Preferred Reporting Items for Systematic Reviews and Meta-Analysis ( PRISMA). The completed PRISMA checklist is available in *Reporting guidelines.* ^35^ This search of titles and abstracts was limited to articles that were human-focused and published in English and Bahasa Indonesia. Statistical analysis was carried out with Review Manager (RevMan, Cochrane, London, UK) version 5.4.1 with standardized mean difference and risk ratio as a measure of the effect of therapy.

**Table 1.** Database search strategy.

Database	Keywords
**Cochrane Library**	eczema OR atopic in Title Abstract Keyword AND therap* OR treatment in Title Abstract Keyword AND vitamin D in Title Abstract AND children OR child OR paediatrics OR paediatrics AND Clinical trials AND SCORAD
**PubMed**	(((((eczema [MeSH Terms]) OR eczema [Title/Abstract]) OR dermatitis [Title/Abstract]))) AND ((Vitamin D [MeSH Terms]) OR Vitamin D [Title/Abstract])) AND (((treatment [Title/Abstract]) OR therap* [Title/Abstract]) OR therapeutics [MeSH Terms])
**ProQuest**	(ti (eczema* OR dermatitis OR atop*) OR ab (eczema* OR dermatitis OR atop*)) AND ti (children OR paediatrics OR pediatri) OR ab(children OR paediatrics OR pediatri) AND ti (therapy OR treatment) AND (ti (vitamin D) OR ab (vitamin D)).
**www.clinicaltrials.gov**	*vitamin D* AND *Interventional Studies* AND *Atopic Dermatitis* AND *SCORAD* AND *Child*
**Google Scholar**	ti AND ab (efficacy AND vitamin D AND Atopic Dermatitis AND SCORAD AND Child AND Randomized Control Trial)

In studies that include children and adults as participants, we contacted the author to obtain separate data that contained child subjects only. The ethical clearance of this study has been published from the Ethical Committee of Dr. Soetomo General Academic Hospital Surabaya number 0206/LOE/301.4.2/XI/2020. We did not register the protocol.

### Eligibility criteria for inclusion and exclusion

Intervention studies including Randomized Control Trials and Prospective Cohort studies with clinical outcomes measured on a scale in both groups, before and after the intervention were assessed. Inclusion criteria were as follows: (1) studies with age group 0-18 years old and diagnosed as mild, moderate, or severe atopic dermatitis in both females and males; (2) No restriction to the duration of intervention, type of vitamin D, doses used, frequency and route of administration, and clinical outcome measuring scale: SCORAD, EASI (Eczema Area and Severity Index), IGA (Investigator Global Assessment). (3) studies that provided complete data for clinical outcomes. Exclusion criteria were articles that did not provide full text.

### Outcomes of the study

1. Evaluating the outcome of the disease (changes in SCORAD or EASI) in the Vitamin D supplementation groups compared to placebo groups.
2. Calculating the clinical importance of both groups so that the CER (Control Event Rate), EER (Experimental Event Rate) and NNT (Number Needed to Treat) values can be obtained.

### Data extraction and quality analysis

This analysis included all articles that qualified for selection criteria. Two author, ANH and SS extracted data from each included study including author, country, publication year, study population, AD severity, supplementation dose, frequency, route of administration, duration and outcome scale. The clinical outcome was measured by scale: SCORAD, EASI or IGA. We defined the clinical outcomes as follows:

(1) SCORAD: A clinical measurement tool used to calculate the severity of Atopic Dermatitis patients. The lesion area was calculated based on the rule of nine with a value of 0-100. Intensity was measured in a representative area by looking at the form of skin abnormalities that were erythema, edema, oozing or crusts, excoriations, lichenification, and dry skin, and each was assigned a value of 0 if there was no lesion, 1 if the lesion was mild, and 2 if the lesion was moderate and 3 if the lesion was severe, then the scores were summed to get B (0-18). Subjective symptoms were measured by Visual Analog Scale (VAS), calculated on average for every 3 night whether there were symptoms of itching and sleep disturbances, with a score of 0 if there was no itching or sleep disturbances, and 10 for the most severe itching or sleep disturbances. These numbers are summed to give C (0-20). The results of the three parameters were submitted into the formula A5+7B/2+C, then grouped into mild AD (<25), moderate AD(25-50) and severe AD(>50) categories. ^13^
(2) EASI: an instrument used by examiners (doctors, dermatologists) to quantify lesion progression and severity of AD patients, by assessing the extent of the disease at four body sites (head/neck, trunk including genitalia, superior and inferior extremities) and measures four clinical signs: (1) erythema, (2) induration/papulation, (3) excoriation, and (4) lichenification each on a scale of 0 to 3. The score can then be divided into 0 (clean), 0.1-1.0 (nearly clean), 1.1-7 (mild), 7.1-21 (moderate), 21.1-50 (severe), 50,1-72.0 (very severe). EASI confers a maximum score of 72. ^13^
(3) IGA: an instrument used to assess overall disease severity at one given time point, and it consists of a 6-point severity scale from clear to very severe disease (0 = clear, 1 = almost clear, 2 = mild disease,3 = moderate disease, 4 = severe disease and 5 = very severe disease). IGA uses clinical characteristics of erythema, infiltration, papulation, oozing and crusting as guidelines for the overall severity assessment. ^14^

The covidence quality assessment template was customized for this study and the quality of each study was assessed by three authors (ANH, SS and DWS) independently by using the Centre for Evidence-Based Medicine’s RCT (Randomized Control Trial) worksheet, by conducting a critical appraisal to determine validity, importance and applicability. Validity was assessed based on Recruitment, Allocation and Measurement Blinding Outcome. Importance was assessed based on clinical data as well as statistical data. Applicability was assessed by answering several questions related to the author’s setting ( Table 2). Critical appraisal for prospective (cohort) study was conducted using critical appraisal skill programme worksheet ( Table 3). Another author resolved any disagreement between them (CRSP, DMI, and DD). Quality analysis of the interventional studies had showed three studies scoring randomized double-blind clinical trials with adequate randomization and blinding. These were Refs. 15, 16 and 17. The other study did not mention randomization but confirmed the blinding of both participants and researchers. ^18^

**Table 2.** Critical appraisal of included interventional studies in systematic review using Randomized Control Trial (RCT) worksheet.

		Camargo, 2014 ^16^	Galli, 2015 ^21^	Lara-Corrales, 2018 ^15^	Sanchez-Armendariz, 2018 ^17^	Earlia, 2020 ^18^	Mansour, 2020 ^22^
Recruitment	Randomization	Yes	Unclear	Yes	Yes	Unclear	Yes
	Similarity	Yes	Yes	Yes	Yes	Yes	Yes
Allocation	Treated equally	Yes	Yes	Yes	Yes	Yes	Yes
	Minimum loss to follow up	Yes	Yes	Yes	Yes	Yes	Yes
Measurement blinding outcome		Yes	Yes	Yes	Yes	Yes	Yes
Importance	Clinical	+	-	-	+	+	-
	Statistical	p=0.04	P=0.5	p=0.07	p=0.02	P<0.001	p=0.039
Applicability		Yes	Yes	Yes	Yes	Yes	Yes

**Table 3.** Critical appraisal of included cohort studies in systematic review.

	Filippo, 2015 ^23^	Raj 2020 ^24^
Representative of the population	Yes	Yes
Methods for exposure objective and consistent	Yes	Yes
Subjects/outcome accessor blinded	Unclear	Unclear
Sufficiency of follow up	Unclear	Yes
Overcoming confounding factor	Yes	No
Importance	Yes	Yes
Applicability	Yes	Yes

**Figure 1.**
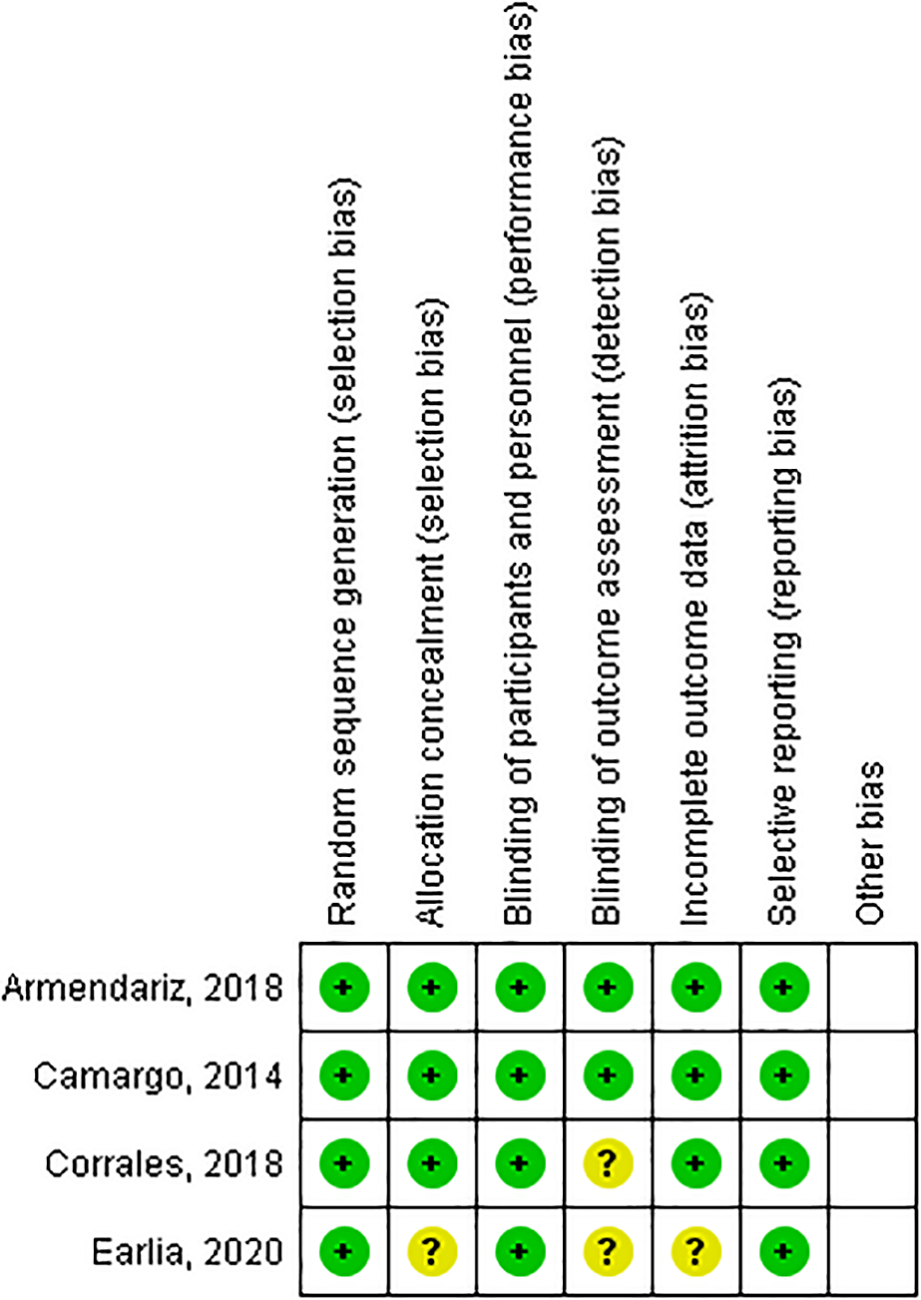
Risk of bias summary: review authors' judgements about each risk of bias item for each included study.

The risk of bias in RCT studies was assessed with The Cochrane Collaboration's tool for assessing risk of bias ^19^ by ANH, SS and DWS, then we discussed the outcome until we were in agreement. The assessment results were categorized as “yes” for low-risk bias, “unclear”, and “no” for high-risk bias ( Figure 1). Cohort studies was assessed with Newcastle-Ottawa Scale (NOS) ^20^ and comprised several items including comparability of the groups (2 points), and ascertainment of exposure (3 points). Each study was interpreted to be low quality (scores <4), moderate quality (scores of 5–6), or high quality (scores ≥7) that was shown in Table 4.

**Table 4.** Quality analysis of included studies using Newcastle Ottawa Quality Assessment Form for Cohort Studies.

*Study*	*Criterion Score*		
	*Selection*	*Comparability*	*Exposure*
**Filippo, 2015** ^23^	★★★	★★	★★
**Raj, 2020** ^24^	★★★	★★	★★

### Statistical analysis

We performed the data with Review Manager (RevMan, Cochrane, London, UK) version 5.4.1. Three authors, DWS, IC, and SA conducted statistical analysis and presented the result in a forest plot and funnel plot. Statistical analysis was done by calculating the standardized mean differences (SMDs), with 95% CIs, of *pre-* and *post-*intervention in both groups, and the standard deviation of each study, and was also calculating risk ratio (RR), with 95% CIs, by counting the number of events in each group with a dichotomy table ( Table 5). Significance of RRs was determined using the Z test (p<0.05 was considered statistically significant). We assessed the heterogeneity among the studies using *I* ^2^ (considered heterogeneity existed if *I* ^2^ > 25%), then Random Effect Model was adopted. For publication bias, we used funnel plot and it can be seen that the four studies are distributed symmetrically, that is, the distribution of research is balanced on the left and right of the centre line boundary. This means that there is no potential for publication bias regarding the conclusions.

**Table 5.** Dichotomy table of studies that provide every subject’s outcome measured on a scale, before and after supplementation in both groups.

	Vitamin D			Placebo		
	Not cure	Cure	Total	Not cure	Cure	Total
**Camargo, 2014** ^16^	25	32	57	27	20	47
**Sanchez-Armendariz, 2018** ^17^	3	16	19	6	18	24
**Earlia, 2020** ^18^	2	13	15	15	0	15

## Results

570 articles were initially retrieved, and the results of the evaluation of duplicate articles by title showed 157 articles with similar titles and were subsequently excluded from this study. The next evaluation was carried out by reviewing the title of each piece of literature that had been searched based on keywords. There were 11 literatures by excluding 402 literature that was irrelevant with the study design. We did further evaluation of 11 kinds of literature based on eligibility criteria, critical appraisal, and quality assessment, and excluded two articles with subject included aged > 18 years old and one article with non-AD subjects as a comparison group. Qualitative synthesis then resulted two studies that could not be included in the meta-analysis due the fact that no standard deviation was reported. ^21^^,^^22^ A further two studies were also excluded due to the study design which was single-arm cohort ^23^^,^^24^ so that the final results were four articles which were then analysed in this study.

**Figure 2.**
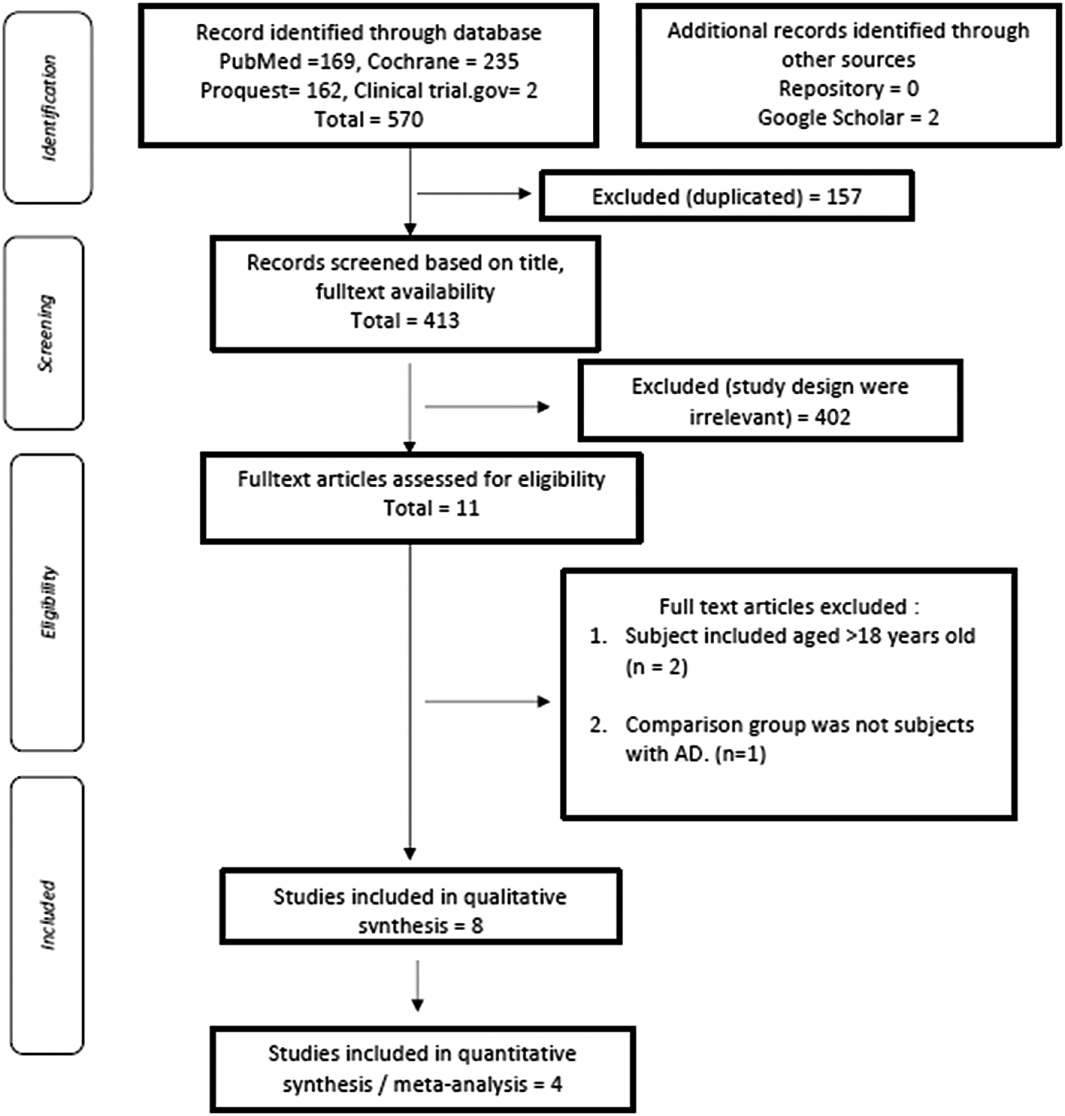
PRISMA flow diagram to show results of the search process and inclusions/exclusions.

Four articles were included in meta-analysis, as described in Figure 2. The years of publication for all studies were ranging from January 2010 to October 2020. Three studies were conducted with paediatric participants only ^15^^,^^16^^,^^18^ and one study was conducted with adult and paediatric participants. ^17^ There were different doses and durations in supplementing vitamin D among studies. One study reported that vitamin D supplementation did not significantly improve the severity of the disease, ^15^ but the other three studies reported otherwise. This study only included AD participants with deficiency or insufficiency status of serum vitamin D. We summarized all studies including population, sample size, intervention, and mean difference outcome of both groups (Vitamin D and placebo groups). All outcomes listed as positive (p<0.05) or negative (p>0.05) are shown in Table 7.

### Effect of vitamin D supplementation in paediatric AD patients

**Figure 3.**
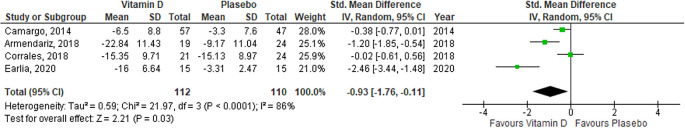
Forest plot for meta-analysis of role of vitamin D supplementation in atopic dermatitis severity.

Four randomized controlled trials assessed the efficacy of vitamin D supplementation. The characteristics of the included studies are summarized in Table 6. Three studies measured the SCORAD indexes, whereas only one of the included studies assessed the efficacy of vitamin D supplementation by using EASI. One study used both adults and children as a participant, so we contacted the author to obtain the data associated with the children only. A meta-analysis of four trials showed that the SCORAD index and EASI score decreased significantly after vitamin D supplementation (standardized mean difference = -0.93, 95% CI = -1.76 to -0.11). We observed statistical heterogeneity among the studies ( *I* ^2^ > 25%; Figure 3). We also assessed the potency of the publication bias in those included studies with funnel plot and the result was symmetrical indicated that there was no potency of the publication bias in the four included studies.

**Table 6.** Characteristics and outcome of included studies.

Author	Country	Dose, frequency and duration	Study design	Mean age (years)	Outcome scale	AD severity	Supplementation giving method	Study population	Result
Camargo *et al*., 2014 ^16^	Mongolia	Vitamin D _3_ 1000 IU/day for 1 month	*RCT*	9	EASI	Mild, moderate, severe	orally	Paediatric patients with winter-associated AD, EASI between 10-72, based on randomization with a random number generator.	Vitamin D supplementation showed clinical improvement
Lara-Corrales *et al*., 2018 ^15^	Canada	Vitamin D 2000 IU/day for 3 months	*RCT*	7.4	SCORAD	Mild, moderate, severe	orally	Paediatric patients with AD, with vitamin D deficiency or insufficiency status	Vitamin D supplementation did not significantly improve severity
Sanchez-Armendariz *et al*., 2018 ^17^	Mexico	Vitamin D _3_ 5000 IU/day for 3 months	*RCT*	12.6	SCORAD	Mild, moderate, severe	orally	Paediatric and adult patients with AD according to the criteria of Hanifin Rajka, which were randomized (simple randomization) with a software.	Vitamin D3 can be considered as adjuvant therapy in AD
Earlia *et al*., 2020 ^18^	Indonesia	Vitamin D 600 IU/day for 1 month	*RCT*	5	SCORAD	Mild, moderate, severe	orally	Paediatric patients with AD who seek for treatment at the Dermatology Clinic for a certain period	Vitamin D supplementation for 1 month was more effective in reducing the severity of AD in children than standard therapy.

Note: RCT, Randomized Control Trial; EASI, Eczema Area Severity Index; SCORAD, Scoring Atopic Dermatitis; AD, Atopic Dermatitis.

**Table 7.** Summary of the included studies.

Author	n-(experimental group)	n-(control group)	Mean difference of intervention group (pre and post Vitamin D supplementation); standard deviation	Mean difference of intervention group (pre and post placebo supplementation); standard deviation	Duration	*p value*	Other outcomes	Adverse effect
Camargo *et al*., 2014 ^16^	57	47	-6,5 (8,8)	-3,3 (7,6)	1 month	0,04	IGA Score in the experimental group was smaller than the placebo group	None
Lara-Corrales *et al*., 2018 ^15^	21	24	-15,35 (9,71)	-15,13 (8,97)	3 months	0,07	Patients with severe AD have low 25(OH) D levels	None
Sanchez-Armendariz *et al*., 2018 ^17^	19	24	-22,84 (11,43)	-13,45 (11,04)	3 months	0,02	Serum vitamin D levels in all experimental groups reached >30ng/ml at the end of the study.	Not reported
Earlia *et al*., 2020 ^18^	15	15	-16 (6,64)	-3,31 (2,47)	1 month	<0,001	None	Not reported

Note: n, Number of subject; IGA, Investigator Global Assessment; AD, Atopic Dermatitis; 25(OH) D, 25-hydroxy vitamin D.

### Risk ratio of vitamin D supplementation group

**Figure 4.**
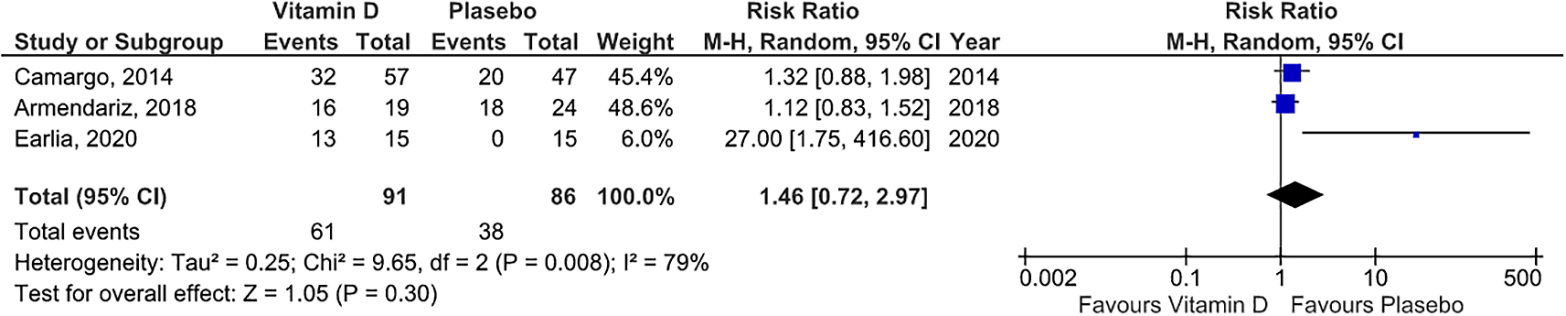
Forest plot for meta-analysis of role of vitamin D supplementation in resulting risk ratio.

We used the three studies that provided raw data so that the risk ratio of those studies could be measured. The forest plot (see *Underlying data* ^35^) showed that statistically, there was no difference risk ratio between vitamin D group and placebo group (risk ratio =1.46 , 95% CI = 0.72 to 2.97). We observed statistical heterogeneity among the studies ( *I* ^2^ > 25%; Figure 4) so Random Effect Model was adopted.

## Discussion

Vitamin D can modulate the innate immune system and also increases the phagocytic ability of immune cells and strengthens the barrier function of epithelial cells. ^8^

An *in vitro* study reported that cathelicidin and defensin (which are antimicrobial-like peptides) increased after vitamin D supplementation. ^25^ Another clinical trial also demonstrated that cathelicidin production could be increased and LL-37 expression could be induced by vitamin D supplementation. Thus, vitamin D could increase antimicrobial activity and external tolerability against pathogens. ^26^

Vitamin D stimulates the production and regulation of skin antimicrobial peptides, such as cathelicidins which exert direct antimicrobial activity and induce host cellular responses to produce cytokine release, inflammation, and angiogenesis, thus, based on the above theory, vitamin D deficiency may predispose to secondary infection in AD patients. ^27^ This is following what was reported by Haridas, Udompataikul *et al*. who found a reduction in *S. aureus* colonization in a paediatric population, as well as Rahmawati *et al*, who have reported that there was a significant difference in the reduction of *S. aureus* colonization after vitamin D3 supplementation in children with AD Refs. 28 and 29.

This theory is following the results of the meta-analysis of our forest plot. Our findings showed a statistically significant difference between the vitamin D supplementation group and the placebo group. In our study, we found high heterogeneity and we assumed that it was caused by variation of the doses and duration. The meta-analysis published by Kim in 2016 ^30^ reported the same results but for the paediatric and adult population, as well as the meta-analysis reported by Haridas in 2018. To our knowledge, our study is the first one that reported vitamin D supplementation efficacy only in children population with AD as the meta-analysis.

The outcome of cure rate is one of the risk ratio, wherein this study the risk ratio calculated is the comparison of the probability of recovered participant between vitamin D and placebo. In the forest plot with risk ratio output, three squares were obtained, each represented 3 studies, with a weight of 45.4%; 48.6%, and 6%. All of these studies have heterogeneity above 50% and p-value <0.05 so that the forest plot used the Random Effect Model as seen from the heterogeneity test results and with the *eyeball test.* Diamond, the result of all studies is on the left side, with a pooled result of 1.46 (CI between 0.72 to 2.97) and touched the vertical line, which means that statistically, there was no difference in cure rate in the vitamin D group and the placebo group. Previously, there were no published meta-analysis with a forest plot with risk ratio outcomes so to our knowledge, our finding is the first meta-analysis with the risk ratio outcome, by point of interest “cure rate” in the experimental group compared to placebo.

The limited number of studies, differences in dose and duration of vitamin D supplementation, differences in race and geographic conditions, dietary variation, sun exposure, as well as differences in measurement variables after vitamin D supplementation administration could be factors that influenced the risk ratio of cure rate, so we expect that more similar studies can be carried out in 1 country thereby reducing the heterogeneity of each study.

In this study, the clinical significance could only be calculated from 3 studies that provided data on the proportion of subjects who recovered and did not recover or had persistent symptoms from the start of the study to the end. As a determination of the criteria for recovery, we had referred to a journal that mentioned MCID (Minimal Clinically Important Difference) in AD, MCID could be described as a clinical improvement that significantly along with reduction of SCORAD of 9 points and EASI by 6 points and IGA score reduced by 1 point. ^31^ From Table 5, Figure 1 it can be calculated that the CER or incidence in the control group (placebo) was 38/86, which is 44%, means that 44% of cases were cured in the group of subjects who were given placebo and EER or the incidence in the experimental group was 61/91, which is equal to 67%, which means that 67% of cases of cure were found in the vitamin D group. ARR (Absolute risk reduction) in both groups was enabled by reducing CER and EER by 23%, and NNT (number needed to treat) was the amount subjects who must be treated at one time to prevent 1 adverse outcome. In these studies, NNT = 4.34 or required 5 subjects to be treated to prevent 1 unwanted event.

From the literature we obtained, only 1 study used 2 phases in the flow of participant/subject selection. This study measured serum vitamin D levels in pediatric AD patients, and only AD patients with insufficiency and deficiency status were included, thereby reducing the possibility of heterogenity in experimental group and control group characters. ^15^ Another study measured the vitamin D levels of all subjects in both the placebo group and the experimental group, and found that 100% of patients given vitamin D3 supplements reached sufficient levels at the end of the study. ^17^ Two other studies did not measure vitamin D levels in subjects either before or after administration of vitamin D supplements, and these two studies used doses that tended to be lower than other studies, and the duration of administration was relatively shorter. ^16^^,^^18^ What is interesting is that the study conducted by Corrales actually showed that vitamin D supplementation did not provide significant clinical improvements in pediatric AD patients. ^15^ In our opinion, the patient's vitamin D3 status at the beginning of the study might be an influencing factor in the patient's clinical improvement. Apart from that, the doses and duration of administration would also have an influence in giving improvement in AD patients.

If toxicity occurs, there will be an increase in 25(OH) D levels which can trigger hypercalcemia by increasing calcium absorption and bone resorption. Hypercalcemia can lead to hypercalciuria, and persistently elevated calcium levels can lead to polyuria and dehydration. ^32^ Vitamin D toxicity is caused by hypercalcemia, which is described by the appearance of symptoms in several organs that can be involved, such as the central nervous system (lethargy, apathy, depression to coma), heart and blood vessels (hypertension, heart rhythm disturbances), gastrointestinal (vomiting), recurrent abdominal pain, anorexia, constipation, and weight loss), and kidney (hypercalciuria is an early symptom, polyuria, polydipsia, nephrocalcinosis, up to life-threatening symptoms such as dehydration and kidney failure requiring haemodialysis). ^33^ The diagnosis of vitamin D toxicity was established based on a detailed examination and history of taking medication, as well as supporting examinations. Laboratory tests show suppression of parathyroid hormone, which results in increased levels of 1,25(OH)2D. ^34^

### Limitations

Dose and duration among studies are not similar, and not all studies have observed vitamin D levels before and after supplementation so it has not been seen whether there is an increase in vitamin D levels that exceeds the limit, which could potentially cause signs of vitamin D toxicity in several organs.

The limitation of our study was that we did not perform sub-group analysis outcome according to the measuring scale and the severity of AD due to the limitations of the studies included, so that the result of our study should be used carefully.

### Suggestions

Further trials with vitamin D3 with the same dose and duration, followed by observation of serum vitamin D levels as an evaluation of the occurrence of side effects.

## Conclusions

Our study has showed that statistically, vitamin D supplementation can improve the outcome of atopic dermatitis in children as assessed by SCORAD, EASI or IGA Score and clinically, vitamin D supplementation can increase the cure rate in AD patients. Observation of side effects and monitoring of 25(OH) D levels in AD patients are required as the toxicity can lead into morbidity. The recommendation of the proper dose of vitamin D supplementation cannot be determined yet because there were no studies with the same dose and duration of administration of the vitamin D supplementation.

## Data availability

### Underlying data

Figshare: Data for Efficacy of vitamin D supplementation on the severity of atopic dermatitis in children: A systematic review and meta-analysis. https://doi.org/10.6084/m9.figshare.19091474.v2. ^35^

This project contains the following underlying data:

- Data untuk forest plot.xlsx (data underlying forest plot).

### Extended data

Figshare: Data for Efficacy of vitamin D supplementation on the severity of atopic dermatitis in children: A systematic review and meta-analysis. https://doi.org/10.6084/m9.figshare.19091474.v2. ^35^

This project contains the following extended data:

- Cochrane.jpeg (Cochrane search strategy).
- PubMed.jpeg (PubMed search strategy).
- ProQuest.jpeg (ProQuest search strategy).
- PRISMA flowchart.pdf

### Reporting guidelines

Figshare: PRISMA checklist for ‘Efficacy of vitamin D supplementation on the severity of atopic dermatitis in children: A systematic review and meta-analysis’. https://doi.org/10.6084/m9.figshare.19091474.v2. ^35^

Data are available under the terms of the Creative Commons Attribution 4.0 International license (CC-BY 4.0).

## References

[1] Kennedy K Heimall J Spergel JM : Advances in atopic dermatitis in 2017. *J. Allergy Clin. Immunol.* 2017;142(6):1740–1747. 10.1016/j.jaci.2018.10.012 30359683

[2] Tsai TF Rajagopalan M Chu CY : Burden of atopic dermatitis in Asia. *J. Dermatol.* 2019 Oct;46(10):825–834. 10.1111/1346-8138.15048 31436343

[3] Wang SS Hon KL Kong AP-s : Vitamin D deficiency is associated with diagnosis and severity of childhood atopic dermatitis. *Pediatr. Allergy Immunol.* 2014;25:30–35. 10.1111/pai.12167 24383670

[4] Farajzadeh S Reghabatpour L Aflatoonian M : Assessment of serum level of 25-hydroxy-vitamin D in Iranian children with atopic dermatitis, in Kerman city, an area with high sun exposure. *J. Pak. Assoc. Dermatol [Internet].* 2016 Nov. 19[cited 2023 Sep. 7];25(2):96–100. Reference Source

[5] Kim J Kim BE Leung DYM : Pathophysiology of atopic dermatitis: Clinical implications. *Allergy Asthma Proc.* 2019;40(2):84–92. 10.2500/aap.2019.40.4202 30819278 PMC6399565

[6] Borzutzky A Camargo CA Jr : Role of vitamin D in the pathogenesis and treatment of atopic dermatitis. *Expert. Rev. Clin. Immunol.* 2013;9(8):751–760. 10.1586/1744666X.2013.816493 23971753

[7] Kim BE Leung DYM : Significance of Skin Barrier Dysfunction in Atopic Dermatitis. *Allergy, Asthma Immunol. Res.* 2018;10(3):207–215. 10.4168/aair.2018.10.3.207 29676067 PMC5911439

[8] Sassi F Tamone C D'Amelio P : Vitamin D: Nutrient, Hormone, and Immunomodulator. *Nutrients.* 2018;10(11):1656. 10.3390/nu10111656 30400332 PMC6266123

[9] Martens P-J Gysemans C Verstuyf A : Vitamin D’s Effect on Immune Function. *Nutrients.* 2020;12(5):1248. 10.3390/nu12051248 32353972 PMC7281985

[10] Hattangdi-Haridas SR Lanham-New SA Wong WHS : Vitamin D Deficiency and Effects of Vitamin D Supplementation on Disease Severity in Patients with Atopic Dermatitis: A Systematic Review and Meta-Analysis in Adults and Children. *Nutrients.* 2019;11(8):1854. 10.3390/nu11081854 31405041 PMC6722944

[11] Huang CM Lara-Corrales I Pope E : Effects of Vitamin D levels and supplementation on atopic dermatitis: A systematic review. *Pediatr. Dermatol.* 2018;35(6):754–760. 10.1111/pde.13639 30284328

[12] Scragg R : Emerging Evidence of Thresholds for Beneficial Effects from Vitamin D Supplementation. *Nutrients.* 2018;3;10(5):561. 10.3390/nu10050561 29751504 PMC5986441

[13] Lara-Corrales I Bergman JN Landells I : Approach to the Assessment and Management of Pediatric Patients With Atopic Dermatitis: A Consensus Document. Section I: Overview of Pediatric Atopic Dermatitis. *J. Cutan. Med. Surg.* 2019;23(5_suppl):3S–11S. 10.1177/1203475419882049 31692379

[14] Rehal B Armstrong A : Health Outcome Measures in Atopic Dermatitis: A Systematic Review of Trends in Disease Severity and Quality-of-LifeInstruments 1985–2010. *PLoS One.* 2011;6(4):e17520. 10.1371/journal.pone.0017520 21533286 PMC3076368

[15] Lara-Corrales I Huang CM Parkin PC : Vitamin D Level and Supplementation in Pediatric Atopic Dermatitis: A Randomized Controlled Trial. *J. Cutan. Med. Surg.* 2019;23(1):44–49. 10.1177/1203475418805744 30336685

[16] Camargo CA Jr Ganmaa D Sidbury R : Randomized trial of vitamin D supplementation for winter-related atopic dermatitis in children. *J. Allergy Clin. Immunol.* 2014;134(4):831–835.e1. 10.1016/j.jaci.2014.08.002 25282565

[17] Sanchez-Armendáriz K García-Gil A Romero CA : Oral vitamin D3 5000 IU/day as an adjuvant in the treatment of atopic dermatitis: a randomized control trial. *Int. J. Dermatol.* 2018;57(12):1516–1520. 10.1111/ijd.14220 30238557

[18] Earlia N Maulida M Hidayati A : Pengaruh Pemberian Vitamin D Terhadap Perbaikan Gejala Klinis Pada Penderita Dermatitis Atopik Di Poliklinik Kulit Kelamin RSUD Dr. Zainoel Abidin Banda Aceh Tahun 2018: Uji Klinis Ketersamaran Ganda. *J. Med. Sci.* 2020;1(1):31–37.

[19] Altman DG Higgins JPT : Assessing risk of bias in included studies. Higgins JPT Green S , editors. *Cochrane handbook for systematic reviews of intervention.* London: John Wiley & sons;2008; p.196.

[20] Stang A : Critical evaluation of the Newcastle-Ottawa scale for the assessment of the quality of nonrandomized studies in meta-analyses. *Eur. J. Epidemiol.* 2010;25(9):603–605. 10.1007/s10654-010-9491-z 20652370

[21] Galli E Rocchi L Carello R : Serum Vitamin D levels and Vitamin D supplementation do not correlate with the severity of chronic eczema in children. *Eur Ann Allergy Clin Immunol.* 2015;47(2):41–47. 25781193

[22] Mansour NO Mohamed AA Hussein M : The impact of vitamin D supplementation as an adjuvant therapy on clinical outcomes in patients with severe atopic dermatitis: A randomized controlled trial. *Pharmacol. Res. Perspect.* 2020;8(6):e00679. 10.1002/prp2.679 33145984 PMC7609811

[23] Di Filippo P Scaparrotta A Rapino D : Vitamin D Supplementation Modulates the Immune System and Improves Atopic Dermatitis in Children. *Int. Arch. Allergy Immunol.* 2015;166:91–96. 10.1159/000371350 25791938

[24] Raj KAP Handa S Narang T : Correlation of serum vitamin D levels with severity of pediatric atopic dermatitis and the impact of vitamin D supplementation on treatment outcomes [published online ahead of print, 2020 Oct 12]. *J. Dermatolog. Treat.* 2020:1–4. 10.1080/09546634.2020.1818677 32885699

[25] Wang TT Nestel FP Bourdeau V : Cutting edge: 1,25-dihydroxyvitamin D3 is a direct inducer of antimicrobial peptide gene expression. *J. Immunol.* 2004;173:2909–2912. 10.4049/jimmunol.173.5.2909 15322146

[26] Leung DY Boguniewicz M Howell MD : New insights into atopic dermatitis. *J. Clin. Invest.* 2004;113(5):651–657. 10.1172/JCI21060 14991059 PMC351324

[27] Umar M Sastry KS Al Ali F : Vitamin D and the Pathophysiology of Inflammatory Skin Diseases. *Skin Pharmacol. Physiol.* 2018;31(2):74–86. 10.1159/000485132 29306952

[28] Udompataikul M Huajai S Chalermchai T : The Effects of Oral Vitamin D Supplement on Atopic Dermatitis: A Clinical Trial with Staphylococcus aureus Colonization Determination. *J. Med. Assoc. Thail.* 2015;98(9):S23–S30. 26817206

[29] Rahmawati YW Zulkarnain I Listiawan MY : Pengaruh Vitamin D3 pada Dermatitis Atopik Anak di Indonesia. *Berkala Ilmu Kesehatan Kulit dan Kelamin.* 2019;31(2):123–129. 10.20473/bikk.V31.2.2019.123-129

[30] Kim MJ Kim SN Lee YW : Vitamin D Status and Efficacy of Vitamin D Supplementation in Atopic Dermatitis: A Systematic Review and Meta-Analysis. *Nutrients.* 2016;8(12):789. 10.3390/nu8120789 27918470 PMC5188444

[31] Schram ME Spuls PI Leeflang MMG : EASI, (objective) SCORAD and POEM for atopic eczema: responsiveness and minimal clinically important difference. *Allergy.* 2012;67(1):99–106. 10.1111/j.1398-9995.2011.02719.x 21951293

[32] Vogiatzi MG Jacobson-Dickman E DeBoer MD : Drugs, and Therapeutics Committee of The Pediatric Endocrine Society. Vitamin D supplementation and risk of toxicity in pediatrics: a review of current literature. *J. Clin. Endocrinol. Metab.* 2014;99(4):1132–1141. 10.1210/jc.2013-3655 24456284

[33] Jain D Manoj A Patel G : Hypervitaminosis D: A Rare Pediatric Case Report. *European J. Biomed. Pharm.* 2019;345(7):66–67. 10.1056/NEJM200107053450115

[34] Marcinowska-Suchowierska E Kupisz-Urbańska M Łukaszkiewicz J : Vitamin D Toxicity-A Clinical Perspective. *Front. Endocrinol. (Lausanne).* 2018;9:550. 10.3389/fendo.2018.00550 30294301 PMC6158375

[35] Hidayati AN Sawitri S Sari DW : Data for Efficacy of vitamin D supplementation on the severity of atopic dermatitis in children: A systematic review and meta-analysis. figshare. *Dataset.* 2022. 10.6084/m9.figshare.19091474.v2 PMC10565422 37829249

