# Supplementary material for: Efficacy of vitamin D supplementation on the severity of atopic dermatitis in children: A systematic review and meta-analysis
Source: F1000Res. 2023 Sep 25;11:274. Originally published 2022 Mar 3. [Version 2] doi: 10.12688/f1000research.106957.2 (PMC10565422; doi:10.12688/f1000research.106957.2)
Supplement: Peer review history

# Reviewer report: Reviewer response for version 2

**DOI:** 10.5256/f1000research.155801.r209122

**Published:** 2023-10-10

**Authors:** Windy Keumala Budianti

**Affiliations:** 1 Department of Dermatology and Venereology, Faculty of Medicine, dr. Cipto Mangunkusumo General Hospital, Universitas Indonesia, Jakarta, Indonesia

Dear editor,

 I have reviewed the new revisions made by the authors and it is appropriate with the questions that I asked. I want to update my status of the submission to "Approved".

 Regards,

 Windy Keumala Budianti

Are the rationale for, and objectives of, the Systematic Review clearly stated?

Yes

Is the statistical analysis and its interpretation appropriate?

Yes

Are sufficient details of the methods and analysis provided to allow replication by others?

Yes

Are the conclusions drawn adequately supported by the results presented in the review?

Partly

Reviewer Expertise:

Dermatology and Venereology, Allergy and Immunology, Dermatosis occupational.

I confirm that I have read this submission and believe that I have an appropriate level of expertise to confirm that it is of an acceptable scientific standard.

# Reviewer report: Reviewer response for version 1

**DOI:** 10.5256/f1000research.117964.r126212

**Published:** 2023-05-24

**Authors:** Windy Keumala Budianti

**Affiliations:** 1 Department of Dermatology and Venereology, Faculty of Medicine, dr. Cipto Mangunkusumo General Hospital, Universitas Indonesia, Jakarta, Indonesia

Vitamin D is a therapy that is often used in several inflammatory skin diseases because it has an immunomodulatory effect. One of the diseases is atopic dermatitis (AD), which in children is a problem that is often faced by dermatologists. The cause of AD is based on the disruption of the skin barrier and impaired regulation of the immune response. However, there are still few evidence-based studies on the benefits or efficacy of vitamin D. The authors attempted to conduct a systematic review and meta-analysis with the aim of studying the effectiveness of vitamin D in pediatric atopic dermatitis using several validated measures, such as SCORAD, EASI, and IGA.

 I appreciate the work the authors have done, but there are a few things that might be added to this article.

1. At the introduction, the prevalence of AD in children was presented, it might be better if data were added on the prevalence of vitamin D deficiency or insufficiency in atopic dermatitis in children, which is a risk factor for AD.
2. The method used starts from a literature search, determining eligible criteria for inclusion and exclusion, data extraction and quality analysis, and statistical analysis using Revman version 5.4.1. is already good and clearly visible on the PRISMA flow chart.
3. As for the results, the data for the duration, dose and improvement of AD on vitamin D administration from the literature varied. The study of Earlia et al. showed that with vitamin D supplementation at a dose of 600 IU / day for 1 month of administration showed significant improvement compared to standard therapy alone. In contrast, the Lara-Corrales et al study using a dose of 2000 IU/day for 3 months did not significantly improve AD severity.
4. Meta-analysis studies show that statistically vitamin D supplementation when compared with placebo improves the severity but not cure rate of atopic dermatitis.
5. The conclusion can be added regarding the results of the meta-analysis related to the risk ratio although the results are not statistically significant.
6. In the discussion section, it can be discussed more deeply about the literature included in the study other than the facts that are generally known.

Are the rationale for, and objectives of, the Systematic Review clearly stated?

Yes

Is the statistical analysis and its interpretation appropriate?

Yes

Are sufficient details of the methods and analysis provided to allow replication by others?

Yes

Are the conclusions drawn adequately supported by the results presented in the review?

Partly

Reviewer Expertise:

Dermatology and Venereology, Allergy and Immunology, Dermatosis occupational.

I confirm that I have read this submission and believe that I have an appropriate level of expertise to confirm that it is of an acceptable scientific standard, however I have significant reservations, as outlined above.

# Response

**Published:** 2023-05-26

**Authors:** Afif Nurul Hidayati

**Affiliations:** Airlangga University, Indonesia

Dear Dr. Budianti,

 Firstly, we would like to thank you for having our manuscript reviewed thoroughly, and for the helpful advice.

 Based on our study, there were limitations of the data we used.

 Some of our literatures did not measure the level of Vitamin D in their subjects. Therefore, there was some studies showing improvement and otherwise did not show any significant improvement.

 For the conclusion sections, to our knowledge, the supplementation of Vitamin D in atopic dermatitis patients is one of therapy that offer improvement in disease severity in despite of the doses and duration.

 Thank you for the suggestions in your review

 Best regards,

 Afif Nurul Hidayati

# Reviewer report: Reviewer response for version 1

**DOI:** 10.5256/f1000research.117964.r126213

**Published:** 2022-03-21

**Authors:** Prasetyadi Mawardi

**Affiliations:** 1 Department of Dermatology and Venereology, Faculty of Medicine, Dr. Moewardi General Hospital, Sebelas Maret University, Surakarta, Indonesia

This study provides optimal knowledge about the administration of vitamin D supplementation in children with atopic dermatitis. In this study, the results of a meta-analysis of 4 data included in the inclusion criteria had differences in the duration of vitamin D supplementation. Two data were given within a 3 month period, two data were provided within a 1 month period.

 How do authors present their arguments? Although this has become part of the limitations of the study, it is not explained much in the discussion. Can researchers provide appropriate advice on dosing and duration of treatment with vitamin D supplementation? Vitamin D supplementation in children remains to be understood as an adjuvant therapy. The absence of an adequate dose of vitamin D supplementation should be a consideration for clinicians. It is known that in atopic dermatitis there is a filagrin mutation that causes the stratum corneum barrier system to not function properly and there is a second defect in tight junction abnormalities. Even though we know that an excessive increase in vitamin D levels will have a negative impact on the function of filagrin as the main barrier in patients with atopic dermatitis.

Are the rationale for, and objectives of, the Systematic Review clearly stated?

Yes

Is the statistical analysis and its interpretation appropriate?

Yes

Are sufficient details of the methods and analysis provided to allow replication by others?

Yes

Are the conclusions drawn adequately supported by the results presented in the review?

Yes

Reviewer Expertise:

dermatology and venereology

I confirm that I have read this submission and believe that I have an appropriate level of expertise to confirm that it is of an acceptable scientific standard.

# Response

**Published:** 2022-03-28

**Authors:** Afif Nurul Hidayati

**Affiliations:** Airlangga University, Indonesia

Dear Dr. Mawardi,

 We would like to firstly express our gratitude for having our manuscript reviewed thoroughly, and we would like to thank you for the helpful advice.

 Currently, there is no guideline for recommended dose and duration of vitamin D administration that can be given to patients. However, in several studies, it is stated that patients with AD have low serum vitamin D levels, so some doctors use the dosage recommendations from the AAP (American Association of Pediatrics) and several health organizations.

 In children aged 0-6 months, the maintenance dose for vitamin D administration is 400 IU with an upper limit of 2000 IU/day if including patients at risk of vitamin D deficiency. The largest group of children aged 11-18 years is recommended to give a dose of vitamin D 400 IU by the AAP and 600-1000 IU by the Endocrine society and 4000 IU at the upper limit dose. Therapeutic doses in patients with vitamin D deficiency or insufficiency range from 1000 IU to more than 5000 IU per day, according to age group.
